# Patients with juvenile idiopathic arthritis in clinical remission with positive power Doppler signal in joint ultrasonography have an increased rate of clinical flare: a prospective study

**DOI:** 10.1186/s12969-017-0208-7

**Published:** 2017-11-13

**Authors:** Vanessa Bugni Miotto e Silva, Sônia de Aguiar Vilela Mitraud, Rita Nely Vilar Furtado, Jamil Natour, Claudio Arnaldo Len, Maria Teresa de Sande e Lemos Ramos Ascensão Terreri

**Affiliations:** 1 Pediatric Rheumatology Unit, Allergy, Immunology and Rheumatology Division, Pediatric Department, Universidade Federal de São Paulo/Escola Paulista de Medicina (UNIFESP/EPM), Borges Lagoa Street, 802, Vila Clementino, São Paulo, SP Zip code 04038-001 Brazil; 2 Imaging Diagnostics Department, UNIFESP/EPM, São Paulo, Brazil; 3 Rheumatology Division, Department of Medicine, UNIFESP/EPM, São Paulo, Brazil

**Keywords:** Juvenile arthritis, Synovitis, Ultrasonography, Power Doppler, Flare

## Abstract

**Background:**

Ultrasonography (US) studies carried out on joints of juvenile idiopathic arthritis (JIA) patients in clinical remission demonstrate the presence of subclinical synovitis. The significance of subclinical synovitis and the positive power Doppler (PD) signal on US in JIA in clinical remission is not well understood. The objectives of this study were to assess whether the changes detected by US in patients with JIA in clinical remission can predict disease flare and to evaluate factors associated with flare and joint damage over 30 months of follow-up.

**Methods:**

A prospective study was performed with clinical and ultrasound evaluation in 34 joints of JIA patients in clinical remission. Clinical evaluation including physical exam, functional capacity and inflammatory markers was performed at baseline and every six months thereafter, for a total period of 30 months. US evaluation included presence of synovitis, PD signal and erosion at baseline and every 12 months thereafter. Subclinical synovitis was defined when there was synovitis with or without positive PD signal in US joints of patients in clinical remission. Flare was defined as any joint presenting clinical arthritis requiring therapy modification.

**Results:**

We evaluated a total of 35 patients, 28 (80%) girls, 14 (40%) persistent oligoarticular subtype, 12 (34.3%) oligoarticular extended and 9 (25.7%) polyarticular and 26 (74.3%) in remission on medication. Twenty (57.1%) patients flared. The risk of flare was five times higher in patients with positive PD signal and 14 times higher in patients in remission on medication. Regarding the assessment of joints after 6 months and 12 months of US evaluation, 70/3162 (2.2%) joints and 80/2108 (3.8%) joints flared, respectively. Joints with subclinical synovitis with positive PD signal flared more after 6 and 12 months. Twenty five of 2108 (1.2%) joints showed erosion over time. Joints with subclinical synovitis with or without positive PD signal showed more erosion.

**Conclusions:**

Patients in remission on medication with subclinical synovitis with positive PD signal on US have a higher risk of flare, therefore they should be monitored closely during treatment. In the same way, joints with subclinical synovitis with or without positive PD signal should be monitored due to the risk of flare and joint damage.

## Background

Juvenile idiopathic arthritis (JIA) is the most common chronic rheumatic disease in childhood, causing disability and reduced quality of life. It is a heterogeneous condition, characterized by periods of activity and clinical remission [1–4].

Joint ultrasonography (US) is a promising tool for diagnostic, prognostic and treatment efficacy evaluation in JIA patients. It can improve upon the physical examination by providing assessment of specific joints, such as the hips, ankles, midfoot and wrists, it has the potential for early detection of synovitis and determination of JIA activity and flare and it improves accuracy of steroid placement in the joints for intra-articular injections [5–12]. US studies carried out in joints of JIA patients in clinical remission demonstrate the presence of subclinical synovitis [5, 7, 13–20]. Subclinical synovitis detected by US and magnetic resonance imaging (MRI) is common in adults with rheumatoid arthritis (RA) in clinical remission and is associated with structural progression and joint damage [21–25].

The significance of subclinical synovitis and the positive power Doppler (PD) signal on US in JIA in clinical remission is not fully understood. Persistent ultrasound abnormalities may reflect residual inflammatory activity undetected by clinical and laboratory examinations, with increased risk of disease flare and joint damage progression [7, 14, 16, 26]. Magni-Manzoni et al. evaluated the ultrasonographic abnormalities in relapses of patients with JIA in clinical remission and found that none of the parameters were of prognostic value for patients with JIA, contradicting the findings in adults. Different from what is reported in RA, this study found that patients with persistent inactive disease had a greater frequency of PD signal than patients who flared. This shows that further studies should be performed to assess the true value of ultrasound changes – including the PD signal - in relation to JIA activity and remission [17].

The objectives of this study were to evaluate whether changes detected by US in patients with JIA in clinical remission could predict disease flare or joint damage (erosion) and to evaluate possible factors associated with JIA flare and joint damage over 30 months of follow-up.

## Methods

### Population

A longitudinal study was carried out in our Pediatric Rheumatology Unit. We evaluated JIA patients classified according to the criteria of the International League of Associations for Rheumatology (ILAR) [27, 28] and considered in clinical remission by the Wallace’s criteria [29, 30] from August 2010 to October 2013.

Inclusion criteria:

- Oligoarticular or polyarticular JIA
- Clinical remission
- Aged between 5 and 18 years

Exclusion criteria:

- Overlap with other autoimmune rheumatic diseases
- Deformity that could compromise the ultrasonographic evaluations proposed by the study
- Other associated diseases that could compromise the joint evaluation (e.g., diabetes mellitus, hypothyroidism)

### Clinical and laboratory evaluation

Patients were evaluated by an experienced pediatric rheumatologist with more than 20 years of clinical rheumatology practice (MTT), certified by the Pediatric Rheumatology International Trials Organisation (PRINTO) and who was blinded to the US findings, to confirm the clinical remission and assess the progression of the clinical and functional features. The following were evaluated: current age, age of disease onset, disease duration, disease subtype, type of clinical remission (on and off medication) and time on clinical remission.

The following parameters were considered in the clinical assessment:

- Active/limited joint count
- Assessment of functional capacity using the Childhood Health Assessment Questionnaire (CHAQ) [31]
- Physician’s global visual analog scale (VAS) (0–10)
- Parent or patient’s global VAS (0–10)
- Medications used: non-steroidal anti-inflammatory drugs (NSAIDs), corticosteroids, disease-modifying antirheumatic drugs (DMARDs) and biologics
- Need to introduce or increase the dose of therapy

Patients underwent laboratory tests, including complete blood count, erythrocyte sedimentation rate (ESR) (Ves-Matic; reference value: 0–20 mm^3^/h) and C-reactive protein (CRP) (nephelometry and/or agglutination; reference value: <0.8 mg/dL), as well as ophthalmologic assessment to determine the absence of active uveitis. The clinical, laboratory and ophthalmologic evaluations were performed at the beginning of the study and every six months thereafter, for a total period of 30 months for each patient.

### Ultrasonographic evaluation

The ultrasonographic evaluation was performed by an experienced musculoskeletal radiologist (SAVM), who was blinded to the clinical variables, at the beginning of the study and every 12 months thereafter. The estimated time for performing the exam in each child was 20 to 30 min. The gray scale and PD ultrasonographic evaluation was performed using a MyLab™ 60 device (Esaote) with a linear transducer with frequency between 6 and 18 Mega Hertz (MHz) for grayscale and up to 12.5 MHz for PD. A pulse repetition frequency of 500–750 MHz with a low-wall filter was used, and the gain was adjusted so that no signal was seen on the bone surface or below it.

Seventeen joints were evaluated bilaterally: 2nd to 5th metatarsophalangeal joints (MTPs), ankles, knees, hips, elbows, wrists, 2nd to 5th metacarpophalangeal joints (MCPs) and 2nd to 5th proximal interphalangeal joints (PIPs) of the hands, according to the evaluation procedures standardized by the European League Against Rheumatism (EULAR) [32]. The recesses evaluated in each joint were the following:

- 2nd to 5th MTPs: dorsal longitudinal recess
- Ankles: tibiotalar: anterior longitudinal recess and subtalar: medial longitudinal recess
- Knees: suprapatellar longitudinal recess
- Hips: anterior longitudinal recess
- Elbows: anterior and posterior longitudinal recesses
- Wrists: longitudinal dorsal radiocarpal and midcarpal recesses and transverse dorsal distal radioulnar recesses
- 2nd to 5th MCPs: volar and dorsal longitudinal recesses and radial longitudinal recess of the 2nd MCP
- 2nd to 5th PIPs of the hands: volar and dorsal longitudinal recesses

**Fig. 1:**
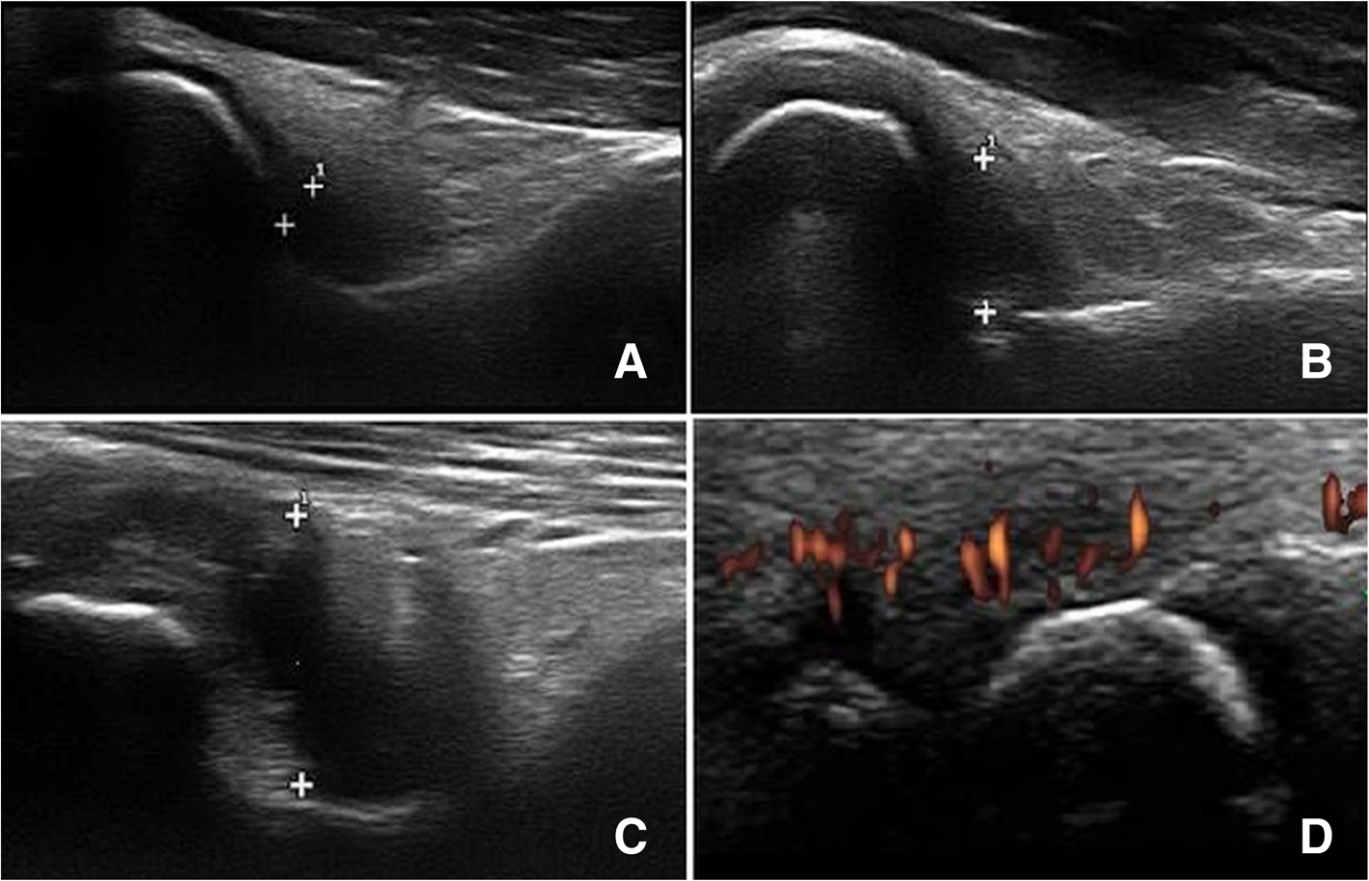
Ultrasonographic parameters. Synovitis in the anterior elbow (**a**. Grade 1; **b**. Grade 2; **c**. Grade 3) and synovial flow in the radiocarpal wrist (**d**. Positive power Doppler signal)

The following ultrasonographic parameters were evaluated, based on the Outcome Measures in Rheumatology (OMERACT)/EULAR definitions [33, 34] (Fig. 1):

A. Synovitis: presence of joint effusion and/or synovial hypertrophy - identified in each joint as an area of ​​hypoechoic or anechoic intra-articular material, compressible and displaceable, with absence of PD signal (joint effusion) or a hypoechoic area of non-displaceable and poorly compressible material, with absence or presence of PD signal (synovial hypertrophy). A semi-quantitative analysis was performed using a score ranging from 0 to 3: 0 – no synovitis; 1 – minimal synovitis in joint recess up to the joint capsule, but without causing it to bulge; 2 – synovitis in the entire joint recess causing bulging of the joint capsule, but without extension to the bone diaphysis; and 3 – synovitis in joint recess with bulging of the joint capsule and extension to at least one bone diaphysis (except for the tibiotalar and subtalar joints). Grades 0 or 1 were considered normal, and grades 2 or 3 were considered definitive pathological changes [35].

B. Blood flow: defined by the presence of PD signal performed only in areas where grade 1 to 3 joint synovitis was detected. A qualitative score, which defined the positive (any degree) or negative PD signal, was used [35].

C. Bone erosion: intra-articular discontinuity of the bone surface seen in two perpendicular planes. Bone erosions were evaluated according to a semi-quantitative scoring system: 0 – regular bone surface; 1 – irregular bone surface without formation of defects seen in two planes; 2 – defect formation on the bone surface seen in two planes; and 3 – bone defect creating extensive bone destruction [35].

Subclinical synovitis was defined in the presence of joint physical examination without arthritis and ultrasonographic examination with grade 2 or 3 synovitis with or without positive PD signal. Erosion was defined in the presence of grade 2 or 3 discontinuity of the bone surfaces.

Information regarding assessment of inter-observer and intra-observer reliability of ultrasound evaluation, as well as the inclusion of a control group of healthy children for comparison with the findings of JIA patients, have been previously reported [19].

Clinical flare of JIA was considered as when any joint exhibited swelling associated with pain, heat and/or limitation on physical examination, requiring a re-introduction or increase of DMARD or intra-articular injection. Flare was assessed 6 and 12 months after each ultrasonographic examination. Joint damage was evaluated by the appearance of erosions in the ultrasound examinations performed at 12 and 24 months.

Categorical variables were analyzed using the chi-squared and Fisher tests. Numerical variables were evaluated using the Mann-Whitney test. To assess the effects of possible factors on the time to flare, the Kaplan-Meier and Cox survival analysis models were used. The log rank test was used to compare categorical variables between the patients who flared and patients who did not over the period analyzed. Cox regression was used to evaluate the effects of numerical variables or of multiple factors simultaneously on the time to flare. The variables significant at 15% were included in the Cox regression with multiple factors. A 5% significance level was adopted for all statistical tests. The statistical programs SPSS 20.0 and STATA 12 were used.

## Results

### Demographic and clinical data

One hundred and eight patients with oligoarticular or polyarticular JIA were regularly evaluated at the beginning of the study, 44 were in clinical remission, on medication or off medication. Of these, four refused to participate in the study, and four had associated comorbidities and were excluded. One patient withdrew from the study after enrollment, and 35 patients were initially evaluated. Thirty-five patients were re-evaluated at 6 months, 32 at 12 months, 30 at 18 and 24 months and 28 at the final 30-month follow-up, due to loss of follow up and were not later scanned. Of the 35 patients at the initial evaluation, 26 (74,3%) were in remission on medication, with a mean time on clinical remission of 1,42 years. No patient had positive rheumatoid factor. The medication used were methotrexate (20 patients), leflunomide (2 patients), cyclosporine (1 patient), methotrexate and biologic (1 patient) and hydroxicloroquine (2 patients). Nine patients were in remission off medication, with a mean remission time of 3 years. Demographic and clinical data from the initial evaluation of all patients are shown in Table 1.

**Table 1:** Demographic and clinical data of all patients (*n* = 35) at the initial evaluation

Mean age ± SD	11.6 ± 3.8 years
Mean age at JIA onset ± SD	4.4 ± 3.2 years
Mean time of JIA duration ± SD	7.1 ± 3.5 years
Females	28 (80%)
Subtype	
Oligoarticular persistent	14 (40%)
Oligoarticular extended	12 (34.3%)
Polyarticular^a^	9 (25.7%)
ANA positive	20 (57.1%)
Previous uveitis	4 (11.4%)
Type of clinical remission	
On medication	26 (74.3%)
Off medication	9 (25.7%)
Mean time on clinical remission ± SD	1.9 ± 2.2 years
CHAQ median (minimum – maximum)	0 (0–0.375)
Global physician’s VAS median (minimum – maximum)^b^	0 (0–1)
Global parents or patient’s VAS median (minimum – maximum)	0 (0–5)
ESR mm^3^/h (mean ± SD)	6.8 ± 4.9
CRP mg/dL (mean ± SD)	0.2 ± 0.15

*SD* standard deviation, *JIA* juvenile idiopathic arthritis, *ANA* antinuclear antibody, *ESR* erythrocyte sedimentation rate, *CRP* C-reactive protein, *CHAQ* Childhood Health Assessment Questionnaire, *VAS* visual analogue scale

^a^No patient had positive rheumatoid factor. ^b^One patient received a Global physician’s VAS of 1 due to joint limitation explained by previous joint damage and not due to active disease at the time of evaluation

Of these 35 patients evaluated, 20 (57.1%) patients had JIA flare during the evaluation period. There was no difference in clinical data and medication use between patients who flared and did not. Demographic and clinical data from the initial evaluation of the patients who flared and patients who did not during the study are shown in Table 2.

**Table 2:** Demographic and clinical data from the initial evaluation of the patients who flared and did not during the study

Total patients (*n* = 35)	Flare (*n* = 20)	No flare (*n* = 15)	*p*
Mean age ± SD (years)	11.5 ± 3.6	11.6 ± 4	0.856‡
Mean age at JIA onset ± SD (years)	3.8 ± 2.8	5.1 ± 3.5	0.298‡
Mean time of JIA duration ± SD (years)	7.6 ± 3.1	6.3 ± 4.1	0.202‡
Females	17	11	0.430^a^
Joint involvement			0.486†
Oligoarticular (Oligoarticular persistent)	7	7	
Polyarticular (Oligoarticular extended + polyarticular)	13 (8 + 5)	8 (4 + 4)	
ANA	12	8	0.693†
Previous uveitis	4	0	0.119^a^
Type of clinical remission			0.129^a^
On medication	17	9	
Mean time on clinical remission ± SD	1.2 ± 0.5 years	2.6 ± 3.2 years	0.064‡
CHAQ median (min – max)	0 (0–0.375)	0 (0–0.375)	0.805‡
Global physician’s VAS median (min – max)	0 (0–1)^b^	0 (0)	0.805‡
Global parents or patient’s VAS median (min – max)	0 (0–4)	0 (0–5)	0.352‡
ESR mm^3^/h mean ± SD	7.5 ± 5.7	6 ± 3.5	0.587‡
CRP mg/dL mean ± SD	0.25 ± 0.15	0.23 ± 0.14	0.730‡

*SD* standard deviation, *JIA* juvenile idiopathic arthritis, *ANA* antinuclear antibody, *ESR* erythrocyte sedimentation rate, *CRP* C-reactive protein, *CHAQ* Childhood Health Assessment Questionnaire, *VAS* visual analogue scale

^a^Fisher’s test, † chi-squared test, ‡ Mann-Whitney test

^b^One patient received a Global physician’s VAS of 1 due to joint limitation explained by previous joint damage and not due to active disease at the time of evaluation

### Ultrasonographic data

Throughout the study, 24 (68.6%) patients had subclinical synovitis (nine with positive PD signal), and 7 had erosion in at least one joint. Subclinical synovitis with or without positive PD signal was more frequent in patients who were older at the initial evaluation (*p* = 0.047), older at the onset of JIA (*p* = 0.036), female (*p* = 0.021), with polyarticular involvement (*p* = 0.002) and shorter remission time (*p* = 0.022). There was no relationship between subclinical synovitis and the JIA follow-up time, positive antinuclear antibodies (ANA), previous uveitis, type of remission, CHAQ score, physician’s or parents’ and patients’ VAS, ESR or CRP. Subclinical synovitis with positive PD signal occurred in patients with shorter remission time (*p* = 0.009). There was no association with the other variables.

### Clinical and ultrasonographic data per joint

Initially ninety-seven ultrasonographic evaluations were performed, for a total of 3298 joints evaluated. Of these, 3162 joints were re-evaluated clinically after 6 months and 2108 joints after 12 months. After 6 and 12 months, 70/3162 (2.2%) and 80/2108 (3.8%) of the joints flared, respectively. The flare time after the ultrasonographic evaluation ranged from 0,25 to 18 months, with a mean of 5.95 months. The mean number of joints affected in each flare episode during the study was one joint per flare (range 1 to 16 joints), and the main joints affected were the knees, ankles and wrists.

Throughout the follow-up, the main joints with subclinical synovitis with or without positive PD signal were the wrists (*n* = 21), ankles (*n* = 13), elbows (*n* = 13), knees (*n* = 7) and small joints of hands and feet (*n* = 15). The main joints with subclinical synovitis with positive PD sign were the wrists (n = 7), knees (*n* = 6) and elbows (*n* = 4).

Joints with subclinical synovitis with or without positive PD signal flared more after 6 and 12 months (Table 3).

**Table 3:** Ultrasonographic data of joints and association with flare after 6 and 12 months of evaluation

	Flare after 6 months (*n* = 70)	No flare after 6 months (*n* = 3092)	*p*	Flare after 12 months (*n* = 80)	No flare after 12 months (*n* = 2028)	*p*
Subclinical synovitis with positive PD signal	5 (7.1%)	14 (0.5%)	<0.001^a^	4 (5%)	10 (0.5%)	0.001^a^
Subclinical synovitis with or without positive PD signal	10 (14.3%)	59 (1.9%)	<0.001^a^	8 (10%)	45 (2.2%)	0.001^a^

*PD* power Doppler

^a^ Fisher’s test

A total of 2108 joints were evaluated for erosion, and 25 (1.2%) exhibited erosion after 12 months. The main joints that presented erosion after 12 months were MCFs (*n* = 12), wrists (n = 6) and hips (*n* = 3). Joints with subclinical synovitis, with or without positive PD signal, showed more erosion during the follow-up of the disease (*p* < 0.001). There was no association between erosion and the isolated positive PD signal (*p* = 1).

### Possible factors associated with JIA flare over the 30-month evaluation

In the Kaplan-Meier survival analysis for related categorical variables, the survival functions were different for patients on medication at the time of flare (p < 0.001). For the other variables, there were no differences between groups (gender *p* = 0.56, JIA subtype *p* = 0.791, antinuclear antibodies *p* = 0.403, subclinical synovitis *p* = 0.694 and PD signal *p* = 0.104). The time leading up to flare was shorter in patients on medication at the time of flare and in patients with a subclinical synovitis with positive PD signal. In the univariate Cox model for the continuous variables, none of the variables was significant for the JIA flare (age at onset, follow-up time, ESR and CRP).

**Fig. 2:**
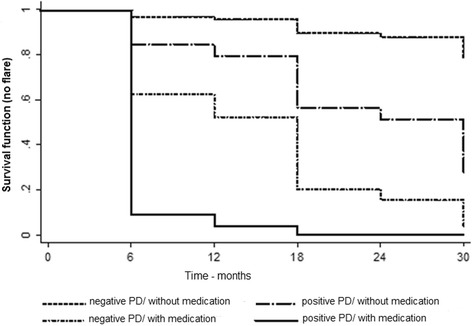
Cox survival function (no flare) for the groups resulting from the combination of PD signal levels and use of medication. PD – power Doppler

In the Cox regression with multiple factors, the presence of subclinical synovitis with positive PD signal (p = 0.104) and the use of medication at the time of flare (*p* < 0.001) were included, and both the subclinical synovitis with positive PD signal (*p* = 0.046) and the use of medication (p < 0.001) were significant. The risk of flare in patients with subclinical synovitis with positive PD signal was five times higher than in patients with subclinical synovitis without positive PD signal on US (hazard ratio (HR) = 5.07, 95% confidence interval (CI) = 1.03 to 24.93). Patients in remission on medication at the time of flare showed a risk of flare 14 times higher than patients off medication (HR = 14.16, 95% CI = 3.22 to 62.31). Figure 2 shows the survival function (no flare) for the four groups resulting from the combination of subclinical synovitis with positive PD signal and use of medication.

## Discussion

In this study, we evaluated whether ultrasonographic changes in patients with JIA in remission can predict flare and the factors associated with the flare. The subclinical synovitis detected by US was not a predictor of flare; however, subclinical synovitis with positive PD signal separately and remission on medication determined an increased risk of JIA flare. The joints with subclinical synovitis with or without positive PD signal flared more over 12 months, and the joints with subclinical synovitis also showed more erosion during the disease follow-up.

Subclinical synovitis with or without positive PD occurred in approximately two-thirds of the patients during the study, being more frequent in females, in patients with polyarticular involvement, who were older at the initial evaluation, who were older at the JIA onset and in patients with shorter remission time. The subclinical synovitis with positive PD signal occurred in patients with shorter remission time. Data from the literature showed a higher frequency of flare in patients with older age at onset, with polyarticular involvement and under methotrexate at the time of evaluation [19].

Over 50% of patients flared during the study period, with no difference in demographic and clinical data between those who flared and those who did not. A study by Magni-Manzoni et al. carried out in patients with JIA in clinical remission found that the patients with persistently inactive disease were younger at disease onset and at the beginning of the study and were more likely to have a positive PD signal than those who flared [17]. In contrast, we observed that joints with subclinical synovitis with or without positive PD signal flared statistically more after 6 and 12 months. A retrospective study by Nielsen et al. showed that in 62 patients with recently diagnosed JIA, the joints with subclinical synovitis had a 29% probability of developing clinical arthritis 6 months after the initial evaluation with US [36].

Subclinical synovitis with or without positive PD was not a predictor of disease flare in our study. Janow et al. evaluated the knees and ankles of patients with JIA using PD US. Of the patients with subclinical synovitis, 21.4% developed active disease within six months [8]. Subclinical synovitis without positive PD signal may not have been related to disease flare in our study because many of these changes may be residual in the joint, not characterizing subclinical activity. On the other hand, the subclinical synovitis with positive PD signal, indicating hypervascularization of the synovial tissue, is a more specific parameter of joint activity [17, 37–39]. We can also conclude that the most suitable definition for subclinical synovitis must consider the positive PD signal because it was the main predictor of JIA flare.

In patients with RA, the positive PD signal predicts short-term flare after clinical remission and is the major predictor of erosive damage [24, 37]. In our study, there was no association between the positive PD signal and the onset of erosions; the joints with subclinical synovitis (with or without positive PD) showed more erosion during the disease. The mechanism and site of onset of erosions in children are different from those of adults, which may explain the different findings of the RA studies [36, 40, 41].

The study by Magni-Manzoni et al. prospectively evaluated the predictive power of ultrasonographic abnormalities (joint effusion, synovial hypertrophy and positive PD signal) in the relapses of patients with JIA in clinical remission. Of the 39 patients, 38.5% flared, and 61.5% remained in remission, but none of the parameters evaluated had prognostic value for the patients. In this study, the patients with persistent inactive disease had a greater frequency of PD signal than patients who flared and the authors suggested that the low grade of the positive PD signal (most joints were positive grade 1 may be the cause of this finding. In addition, patients with sustained remission, who had a greater prevalence of positive PD signal, were younger than patients with synovitis flare [17]. In our study, the positive PD signal, regardless of grade, determined a higher risk of JIA flare, corroborating with the findings of studies on RA [21–25, 42]. In addition, contrary to the findings of Magni-Manzoni et al., in our study all patients – except one who was 7.5 years old – with a positive PD sign were adolescents with a mean age at the beginning of the study of 12.6 years (range 7.5 years to 15.9 years), which strengthens the fact that our findings related to the positive power Doppler signal were actually related to increased synovial blood flow by subclinical disease activity rather than normal intra-articular flow.

Our study also found that the use of medication during clinical remission determined a higher risk of JIA flare. Collado et al. found more ultrasonographic changes in patients with inactive JIA on medication, although the difference was not statistically significant [20]. This finding suggests that patients who are in clinical remission and still on medication have low disease activity but not actual inactivity, which places them at higher risk for disease flare.

This study has some limitations. The number of JIA patients evaluated was small. Also, there was only one pediatric rheumatologist who examined all patients and It would have increased the strength of the study to have had a different evaluator for the clinical evaluation. However, we believe that the extensive experience of our evaluator has been more than sufficient to reduce the evaluation biases that could have occurred. Due to the absence of ultrasonographic standards for children at the time the study was performed, we used criteria defined for adults, which may have affected the interpretation of the results. The OMERACT group recently defined the criteria for joint assessment by US in healthy children and defined the pathological changes in children’s joints [41, 43]. Collado et al. recently systematized the US examination of knee, ankle, wrist and 2nd MCP joints in healthy children of different age groups, leading to the publication of an atlas, including the PD signal findings. In that study, the standardization of the ultrasonographic examination was similar to our method. Furthermore, that study demonstrated that the positive PD signal in the synovial recesses does not occur in healthy children, which reinforces the importance of our results regarding the positive PD signal [44]. A recent study by Lanni et al. found that the lateral recess of the subtalar joint may present more synovitis on US than the medial recess [45]. Unfortunately, in our study, we evaluated only the medial recess of this joint. The ultrasonographic findings were not compared with MRI, which is the current gold standard for joint evaluation. Nevertheless, a study of 59 children with JIA showed that ultrasonographic evaluation of the joints may be comparable to MRI findings [46].

Our study is the first to describe the subclinical synovitis with positive PD signal as a predictor of flare in JIA patients in clinical remission. The current criteria for clinical remission do not include evaluation by imaging, and more studies related to subclinical synovitis with positive PD signal can provide valuable information to optimize the treatment and follow-up of JIA patients.

## Conclusions

Subclinical synovitis with positive PD signal and clinical remission on medication increased risk of JIA flare. Joints that had subclinical synovitis with or without positive PD signal flared more over 12 months. The joints with subclinical synovitis with or without positive PD signal also showed more erosion during disease follow-up. Patients in clinical remission on medication and subclinical synovitis with positive PD signal on US should have the medication withdrawal delayed due to the risk of JIA flare. Joints with subclinical synovitis with or without positive PD signal should be monitored more frequently due to the risk of flare and long-term joint damage.

## Abbreviations

ANA: Antinuclear antibodies
CHAQ: Childhood Health Assessment Questionnaire
CRP: C-reactive protein
DMARDs: Disease-modifying antirheumatic drugs
ESR: Erythrocyte sedimentation rate
EULAR: European League Against Rheumatism
ILAR: International League of Associations for Rheumatology
JIA: Juvenile idiopathic arthritis
MCPs: Metacarpophalangeal joints
MHz: Megahertz
MRI: Magnetic resonance imaging
MTPs: Metatarsophalangeal joints
NSAIDs: Non-steroidal anti-inflammatory drugs
OMERACT: Outcome Measures in Rheumatology
PD: Power Doppler
PIPs: Proximal interphalangeal joints
PRINTO: Paediatric Rheumatology International Trials Organisation
RA: Rheumatoid arthritis
US: Ultrasonography
VAS: visual analog scale
